# A Micro-Force Sensor with Slotted-Quad-Beam Structure for Measuring the Friction in MEMS Bearings

**DOI:** 10.3390/s131013178

**Published:** 2013-09-30

**Authors:** Huan Liu, Shuming Yang, Yulong Zhao, Zhuangde Jiang, Yan Liu, Bian Tian

**Affiliations:** 1 State Key Laboratory for Manufacturing Systems Engineering, Xi'an Jiaotong University, Xi'an 710049, China; E-Mails: LH1981.5@stu.xjtu.edu.cn (H.L.); zhaoyulong@mail.xjtu.edu.cn (Y.Z.); zdjiang@mail.xjtu.edu.cn (Z.J.); ZTlover@stu.xjtu.edu.cn (Y.L.); t.b12@mail.xjtu.edu.cn (B.T.); 2 School of Optoelectronic Engineering, Xi'an Technological University, Xi'an 710032, China; 3 State Key Laboratory of Digital Manufacturing Equipment & Technology, Huazhong University of Science and Technology, Wuhan 430074, China

**Keywords:** micro-force sensor, stress concentration slots, friction measurement

## Abstract

Presented here is a slotted-quad-beam structure sensor for the measurement of friction in micro bearings. Stress concentration slots are incorporated into a conventional quad-beam structure to improve the sensitivity of force measurements. The performance comparison between the quad-beam structure sensor and the slotted-quad-beam structure sensor are performed by theoretical modeling and finite element (FE) analysis. A hollow stainless steel probe is attached to the mesa of the sensor chip by a tailor-made organic glass fixture. Concerning the overload protection of the fragile beams, a glass wafer is bonded onto the bottom of sensor chip to limit the displacement of the mesa. The calibration of the packaged device is experimentally performed by a tri-dimensional positioning stage, a precision piezoelectric ceramic and an electronic analytical balance, which indicates its favorable sensitivity and overload protection. To verify the potential of the proposed sensor being applied in micro friction measurement, a measurement platform is established. The output of the sensor reflects the friction of bearing resulting from dry friction and solid lubrication. The results accord with the theoretical modeling and demonstrate that the sensor has the potential application in measuring the micro friction force under stable stage in MEMS machines.

## 1. Introduction

Micro bearings are very important components for rotating micro-electro-mechanical system (MEMS) devices like turbines, generators, and engines [1]. Compared with macro rotating machines, the MEMS bearings operate at a much higher speed to obtain enough peripheral speed for the rotor blades, which requires a much lower friction. Current research is mainly focused on the fabrication [2,3], slip effect [4,5] or load capacity of MEMS bearings, and barely on the friction. However, investigating the friction of bearings helps to optimize the bearing design and promote the speed of MEMS rotors. Accurate calculation of friction has been a challenge [6–8], while experimental characterization is a cost-effective alternative to the investigation of micro-friction in MEMS bearings.

Due to the small magnitude of the to-be-measured component size and its parameters in the MEMS bearing, conventional methods cannot provide enough accuracy either in installation and measurement [9–11]. For the tests proposed here, a high precision measurement platform is established based on torque balance principle. In this system the friction is transformed into an easily detected force parameter by a force arm, and the sensor to measure the force signal becomes the key device in the system. The requirements defining a desirable force sensor include a high sensitivity to accurately detect the small target forces, a wide measurement range to satisfy different requirements under different lubrication states and an overload protection to avoid unexpected breakage during unsuitable operations.

Many different MEMS force sensors have been developed in relevant literatures, and several configurations have been adopted in force sensing. The membrane structure sensor features a relative large measurement range, but its big supporting stiffness inevitably decreases the measurement sensitivity which limits the sensor's applications. Jordan and Büttgenbach [12] utilized SU-8 resist to develop a membrane force sensor with low bending stiffness, but it is difficult to attach a probe and the low admissible stress of resistance may also limit the available force measurement range. Compared with membrane structures, beam-based structures are more effective. Kulkarni *et al.* reported a force sensor with an optical fiber beam, which is very suitable for monitoring in some harsh environments, but the sensor measurement range is too large and a more flexible beam material is needed when being used in micro friction monitoring [13]. Lu *et al.* reported a piezoresistive-sensor-based micro-force-sensing probe that features good sensitivity and large measurement range [14,15]. Moreover, the sensor can be used in micromanipulation systems if integrated with a micropipette [16]. Beam-based sensors have a greater range of applications, and different target characteristics can be achieved by adjusting the beam dimensions. However, it is a difficult task to make an optimal tradeoff between the measurement range and sensitivity by only changing the beam size, so an improved scheme is needed.

This paper aims at providing a sensing configuration to promote the sensor performance in measurement sensitivity, measurement range and overload protection for MEMS bearing friction measurement systems. The requirements for the sensors are a measurement range of 10 mN and a sensitivity above 0.5 mV/(V·mN), considering the to-be-measured force and experimental conditions in our laboratory. Focused on the target performances, a piezoresistive force sensor with slotted-quad-beam structure is proposed. The sensing structure design, probe choosing and overload protection are described in the design section. Finite element models for the conventional quad-beam structure and proposed structure are established to gain insight into the influence of configuration for the sensor performances. The sensor prototypes are fabricated, packaged and calibrated. A practical friction measurement is also performed to verify the feasibility of utilizing the developed sensor in the measurement system.

## 2. Sensor Design

**Figure 1.**
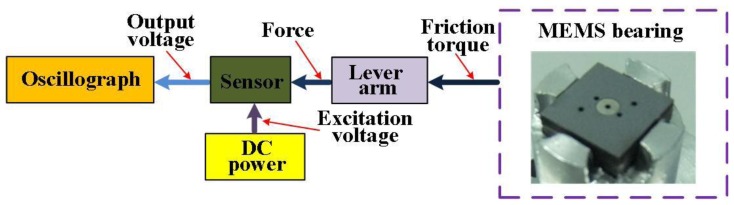
The schematic diagram of the measurement system for friction measurement.

The schematic diagram of the friction measurement system for MEMS bearings is illustrated in Figure 1. The friction usually happens between the rotor and stator in micro bearings, and more details can be found in [17,18]. The to-be-measured friction is transmitted by the lever arm that is attached onto the shaft of bearings. The force is subsequently sensed by the piezoresistive sensor, which should be connected with the lever arm. The oscillograph acquires the output voltage of sensor to build up the variation curve of friction during the rotation of the bearings. For an effective measurement system, the sensor design should meet the following guidelines:

(1) High sensitivity to sensing the micro-force signal.
(2) Good accuracy and linearity.
(3) Overload protection. The silicon-based sensor is fragile, and unpredictable impulsive force during sensor packaging, system assembling or practical measuring may break the sensing structure.
(4) Proper configuration. The sensor will be connected to the lever arm to measure the target force, and a well-designed structure can make the connection more easy and precise.

The first two points are mainly determined by the sensing geometry and structural dimensions, and the latter two are related to the design of sensor components. The probe is often used to transmit the force from a measurement target to the sensing mechanism and overload protection is implemented by bonding a glass or silicon wafer to limit the displacement range of the central mesa. The probe and bonded glass are here selected as the elements in the proposed sensor. In the following part of this section, the investigation of reformative sensing structure will be performed, and the design of force sensor based on the analysis results will be presented with the specific probe and overload protection.

**Figure 2.**
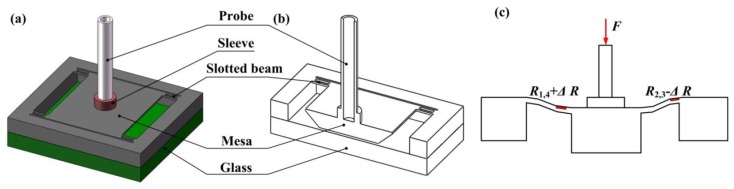
The SQB structure force sensor: (**a**) 3D view; (**b**) cross-sectional view; (**c**) deformation view of the structure under the applied force *F*.

The proposed slotted-quad-beam (SQB) structure is schematically shown in Figure 2. The new scheme incorporates slots for stress concentration into the conventional quad-beam structure and the central mesa is suspended by the slotted beams. Four beams are arranged at the corner of the mesa and the slots are symmetrically settled in the center of beams. The beams are chosen to be relatively short and wide to enlarge the structure stiffness and improve the measurement range. The glass wafer bonded onto the chip bottom is used to provide the overload protection. As Figure 2c shows, when a force *F* is applied to the structure, the slotted beams will be involved in a bending movement together with the vertical motion of central mesa; then the accompanying stress will appear in the beam surface, which changes the resistance of piezoresistors; these changes can be turned into a voltage signal by the Wheatstone bridge, which realizes the transformation from force to voltage.

**Figure 3.**
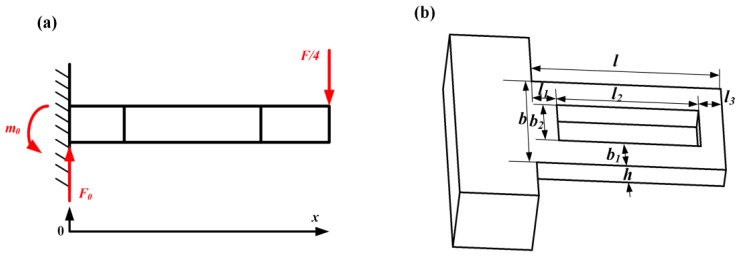
The force analysis and dimensions for SQB structure.

According to the basic principle of mechanics and under the assumption of small deflection, the following equation for a single beam can be derived when a force *F* is applied to the sensor:

(1) $$-EIw\prime \prime (x)=F_{0}x-m_{0}\;(0\leq x<l)$$

where *E* is the Young's modulus of silicon, *I* is the cross-sectional moment, *F*_0_ = *F*/4, and *m*_0_ for the resultant moment at the fixed end of beam, *w*(x) for the beam displacement. Considering the boundary conditions and the denotations of beam and slot dimensions shown in Figure 3, *m*_0_ can be expressed as:

(2) $$m_{0}=\frac{F_{0}}{2}\frac{2b_{1}l_{3}(l+l_{1}+l_{2})+bl_{2}l+2b_{1}l_{1}^{2}}{4b_{1}l_{1}+bl_{2}}$$

where *l*_1_ = *l*_3_ because the slots are located in the middle of the beams. Substituting Equation (2) into Equation (1), the bending moment of the slotted beam can be written as:

(3) $$EIw\prime \prime (x)=\frac{F}{8}l-\frac{F}{4}x$$

Thus, the stress in the slotted beam can be obtained as:

(4) $$\sigma =\frac{EIw\prime \prime (x)y}{I_{z}}=\frac{EIw\prime \prime (x)h}{2I_{z}}$$

Then,

(5) $$\sigma_{x=0}=\frac{3Fl}{4bh^{2}}$$

(6) $$\sigma_{x=l_{1}}=\frac{3Fl_{2}}{8b_{1}h^{2}}$$

**Figure 4.**
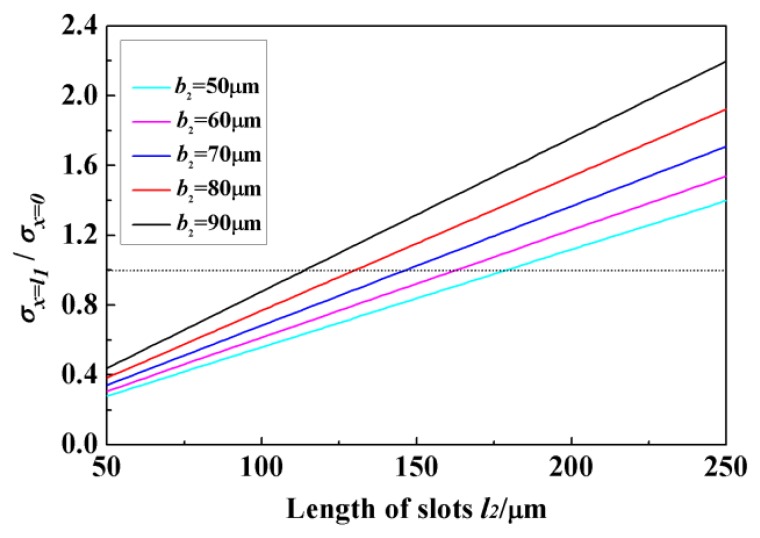
The *σ_x_*_=_*_l_*_1_/*σ_x_*_=0_ value *versus* length of slots *l*_2_.

The stress *σ_x_*_=0_ equals to the maximum stress in normal quad-beam sensor. Figure 4 shows the variation of *σ_x_*_=_*_l_*_1_/*σ_x_*_=0_ versus *l*_2_ for *b*_2_ = 50, 60, 70, 80, and 90 μm when *l* = 260 μm, *b* = 160 μm and *h* = 22 μm. The ratio value is a monotone function of *l*_2_, and the rate of slope changes with the variation of *b*_2_. It can be seen that the stress at the end of slot is increased by enlarging the width and length of slots, and *σ_x_*_=0_ is not always smaller than *σ_x_*_=_*_l_*_1_. The surface value is determined not only by the cross section of the beam but also the location. When the size of slots is relatively small, the enhancing effect is not enough to compensate the location effect, and *σ_x_*_=_*_l_*_1_ < *σ_x_*_=0_; meanwhile, the enhancing effect is increased along the enlargement of slots, and the situation will turn into *σ_x_*_=_*_l_*_1_ > *σ_x_*_=0_, which means the incorporation of slots gives the sensor a better sensitivity. The sensitivity keeps on increasing if the length and width of slots maintain the increase trend. The value of *σ_x_*_=_*_l_*_1_/*σ_x_*_=0_ can be increased by 157% when the slot width varies from 50 μm to 90 μm, and increased by 500% when the length increases from 50 μm to 250 μm. Meanwhile, only evaluating the sensitivity is not a very appropriate approach in the sensor design. Space for piezoresistors and wires should also be considered. Limited by the fabrication conditions in our lab, the width of wires in the sensor chip should be 20 μm at least, and there should be a 5 μm gap between the wires and beam edges. Also, the overlarge slots may also diminish the structural stiffness, influencing the measurement range. Taking all these factors into account, the final size of the slots is determined as: *l*_2_ = 195 μm, *b*_2_ = 70 μm with *σ_x_*_=_*_l_*_1_/*σ_x_*_=0_ = 1.33.

**Figure 5.**
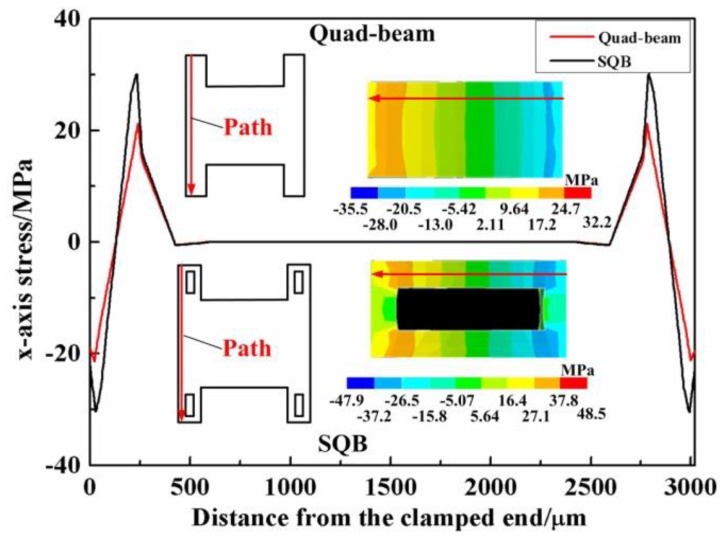
The x-axis stress distribution along the defined paths in two structures.

**Figure 6.**
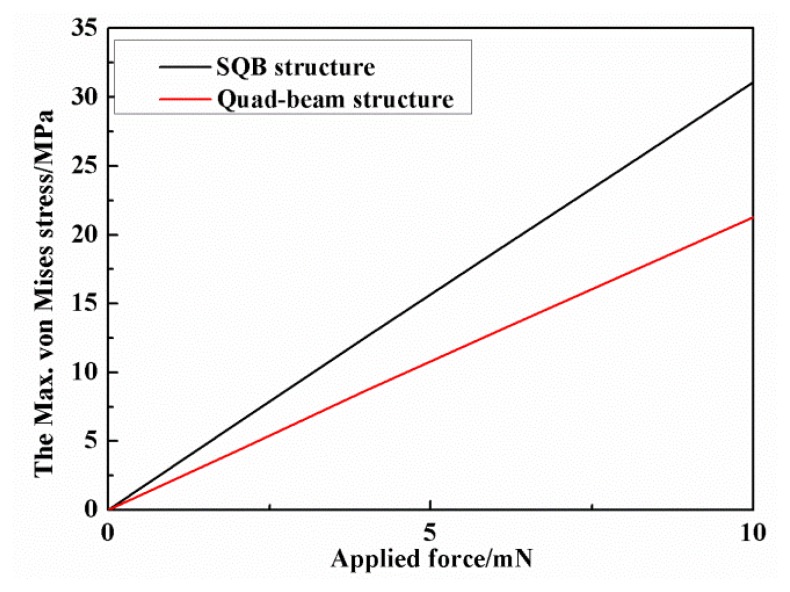
The maximum von Mises stress in SQB and quad-beam structures under different forces.

Finite element simulation is also utilized to verify the proposed structure. Finite elements models for the SQB structure and quad-beam structure are established. The FE simulations are performed by the ANSYS software (version 14.0). The applied forces are symmetrically loaded at the tip of the probe, and the outer frames of sensor are fixed. The material parameters of the silicon are set as Young's modulus *E* = 166 GPa, density ρ = 2331 kg/m^3^ and Poisson's ratio ν = 0.278. Figure 5 shows the simulated x-axis stress in the beams of the two structures under the force of 10 mN. The maximum stress in the beam of SQB structure is 31.09 MPa, while the value in the beam of quad-beam structure is 21.27 MPa. Therefore, the simulated *σ_x_*_=_*_l_*_1_/*σ_x_*_=0_ is calculated to be 1.44 with a deviation of 8.50% when compared with the analytical result. The simulated relationships between these maximum von Mises stresses and applied force are also illustrated in Figure 6. The line slopes indicate that the stress increases linearly with the applied force and the SQB structure has a much larger slope than the conventional quad-beam structure. The ratio of the two slopes is 1.46, which gets a difference of 9.54% when compared with the analytical result (namely the value of *σ_x_*_=_*_l_*_1_/*σ_x_*_=0_). The large deviation between the analytical and simulated results may be caused by the nonlinearity stress distribution near the beam ends [19].

**Figure 7.**
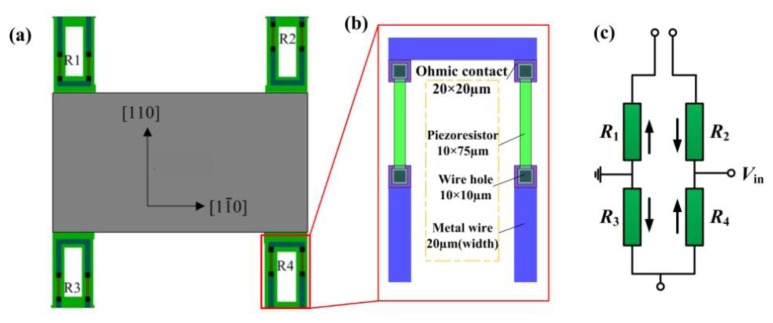
The piezoresistors in the sensor chip: (**a**) the configuration; (**b**) the dimensions; (**c**) the connection of piezoresistors in full Wheatstone bridge circuit.

Consideration is taken of the piezoresistor arrangement to fully sense the force-induced stress on the beam. In this paper, the piezoresistors are located on both sides of the slots along the [110] crystal direction and their dimensions are all shown in Figure 7. The variation of the resistance can be expressed as [20]:

(7) $$\frac{\Delta R}{R}=\frac{1}{2}\pi_{44}\left(\sigma_{\text{l}}-\sigma_{\text{t}}\right)$$

where *R*, *ΔR* are the original and variation resistance of the piezoresistor, *σ*_l_, *σ*_t_ are the stresses in the longitudinal and transverse directions at the central point of the resistors, *π*_44_ is the shearing piezoresistance coefficient [21]. For a full Wheatstone bridge-based sensor with an excitation voltage of *V*_in_, the output voltage *V*_out_ can be obtained as:

(8) $$V_{out}=\frac{1}{2}\pi_{44}\left(\sigma_{\text{l}}-\sigma_{\text{t}}\right)V_{in}$$

The ion concentration is set as 3 × 10^14^ cm^−3^, thus *π*_44_ is 138 × 10^−11^ m^2^ /N [22]. The longitudinal and transverse stresses under different applied force are obtained by FE analysis and *V*_in_ is set as 5 V DC. Therefore, the relationship between applied force *F* (mN) and output voltage *V*_out_ is derived as

(9) $$V_{out}=3.92\times 10^{-3}F$$

Therefore the calculated measurement sensitivity for SQB structure sensor is 0.784 mV/(V·mN), while the value for quad-beam structure is about 0.541 mV/(V·mN). By incorporating the slots into the conventional quad-beam structure, the sensitivity is enhanced by 45%. Another component for the force sensor is the probe. The probe is required to be inflexible and lightweight to eliminate the influence on the sensor. A tubular shape, stainless steel-made probe is chosen here. The inner and outer diameters of the probe are 0.3 mm and 0.45 mm with a length of 10 mm. Moreover, a sleeve is used to enhance the installation precision which is marked red in Figure 2. According to the FE analyses, the acceptable displacement of the central mesa is about 2.6 μm before the sensing structure is broken and the limited force is about 100 mN. Therefore, a Pyrex glass wafer is attached on the bottom of the chip with a gap of 2.6 μm from the bottom of central mesa.

## 3. Sensor Fabrication and Package

### 3.1. Sensor Fabrication

**Figure 8.**
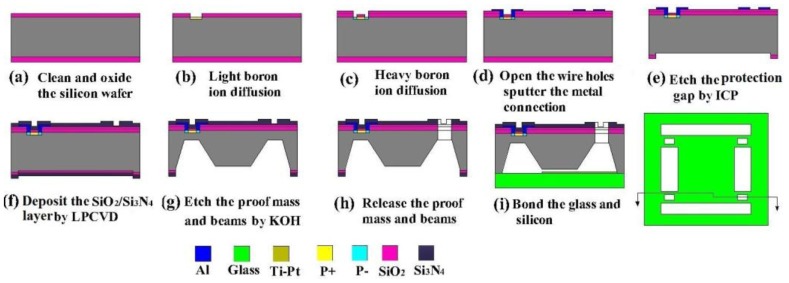
Main fabrication process of the sensor chip.

**Figure 9.**
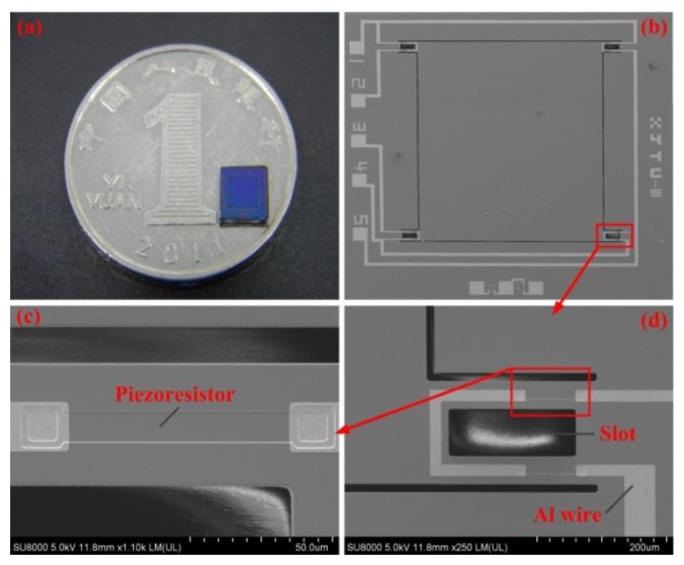
The fabricated sensor chip: (**a**) the sensor chip compared with a coin; (**b**) a photo of the whole chip; (**c**) a detailed SEM photo of one piezoresistor; (**d**) a detailed SEM photo of one slotted beam.

The main fabrication process of the sensor chip is shown in Figure 8. A double-side polished, N-type, [100]-oriented silicon wafer was used as the start material and a total of eight masks were needed. The fabrication began with the thermal oxidization Figure 8a, and the light and heavy boron ion diffusion were conducted after the piezoresistors were patterned at the front side Figure 8b,c; then the interconnections of piezoresistors were realized by Al sputtering and the electrodes were formed at the same time Figure 8d; the inductively coupled plasma (ICP) etching at the back side forms the protection gap between the bottom of central mesa and bonded glass Figure 8e; the deposited Si_3_N_4_ and SiO_2_ were utilized as the protection layer for the following KOH etchant based anisotropic wet etching Figure 8f,g, and the central mesa was shaped by wet etching at the back side; the SQB structure was releaseded by the ICP etching in the front side Figure 8h; a Pyrex glass wafer was bonded onto the bottom of silicon wafer by anodic bonding Figure 8i. A Ti/Pt bi-layer was deposited on the glass wafer to avoid electrostatic adhesion between the mesa and glass during overload protection process. Figure 9 shows the finished sensor chip.

### 3.2. Sensor Package

**Figure 10.**
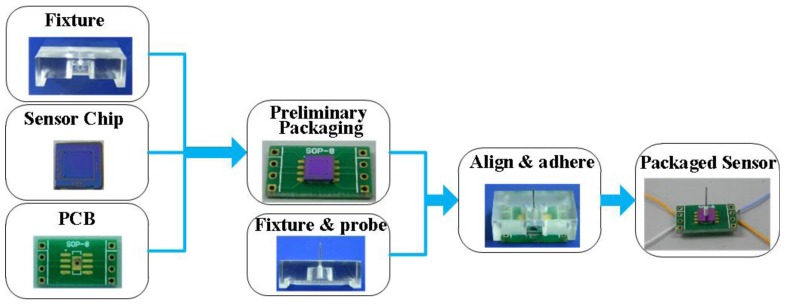
Package of the force sensor.

To guarantee the precision of sensor packaging, a tailor-made organic glass fixture is designed. The fixture has two grooves for the positioning of sensor and a hole for the probe and printed circuit board (PCB). The packaging process is illustrated in Figure 10. The sensor chip and PCB were aligned by the grooves in the organic glass fixture and bonded by adhesive; after that, the probe was put into the hole and adhered onto the central mesa of the sensor chip; finally, the electrical connection between the sensor and instruments was conducted with the golden leads by the wire bonder after necessary thermal solidification.

## 4. Sensor Testing

**Figure 11.**
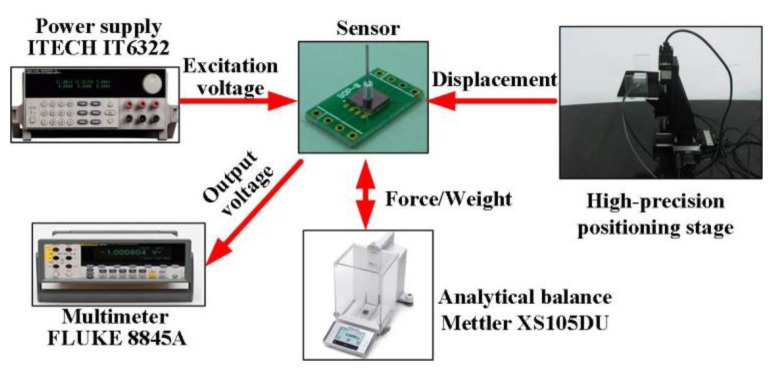
The setup for sensor calibration.

The calibration system, shown in Figure 11, uses an analytical balance and a high-precision positioning stage to generate and display the input force for the sensor; the force can be obtained by multiplying the recorded weight and gravity acceleration (10 m/s^2^). The positioning stage consists of a tri-dimensional positioning stage and a piezoelectric ceramics with the resolution up to nm scale. The tri-dimensional positioning stage adjusts the sensor location, making the tip of sensor probe very close to the sensing plate of balance; then, the piezoelectric ceramics moves the sensor with a nm displacement, vertically pressuring the sensor probe to the balance plate, and the generated force/weight can be read from the balance. The excitation voltage for the Wheatstone bridge in the sensor is provided by the power supply (IT6322, ITECH, Yorba Linda, CA, USA), and the output voltage can be measured by the digital multimeter (8845A, FLUKE, Everett, WA, USA).

**Figure 12.**
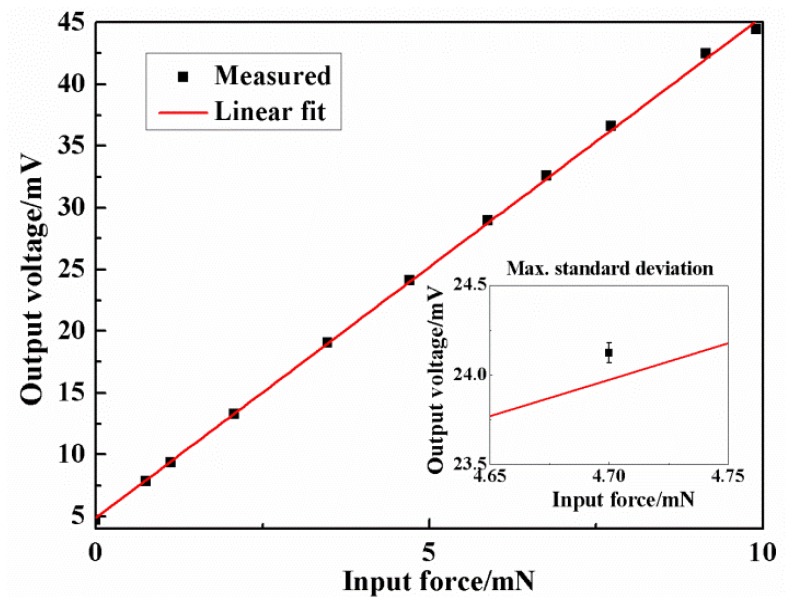
The calibration results.

The static characteristics of the force sensor were tested by the abovementioned calibration system. The excitation voltage for the Wheatstone bridge was 5V DC and the applied force rose from 0 mN to about 10 mN and decreased to 0 mN in one test. The experimental results are shown in Figure 12. In the range of 0–10 mN, the results were fitted with the least square method and the relationship between the applied force *F* (mN) and output voltage *V* (mV) could be expressed as:

(10) V=4.064F+4.874

**Figure 13.**
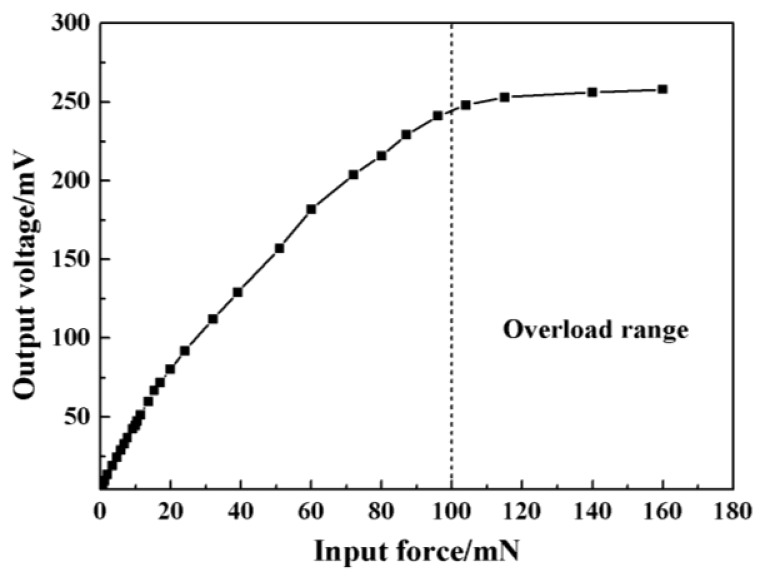
The results of overload protection.

The measured sensitivity was 0.813 mV/(V mN) with a little deviation to the analytical value which might be caused by the fabrication error. The calculated maximum non-linearity was 1.57% FS (full scale) and total measurement accuracy was 2.03% FS. The overload protection was also checked and the results were shown in Figure 13. It could be found that the sensor had a good linearity in the range of 0–10 mN, and the glass began to play the protection role when the applied force was above 100 mN.

Table 1 shows the comparison between the proposed device and previously published designs. It can be seen that the sensitivity decreases when the measurement range is enlarged. The sensor in [23] has the best sensitivity, but the measurement range is too small. The ones presented in [14,24] have a better comprehensive property, but the missing of overload protection restricts the sensor application. Therefore, the proposed sensor obtains a favorable comprehensive property compared to the previous works if considering the sensitivity, measurement range and overload protection.

## 5. Sensor Application

**Figure 14.**
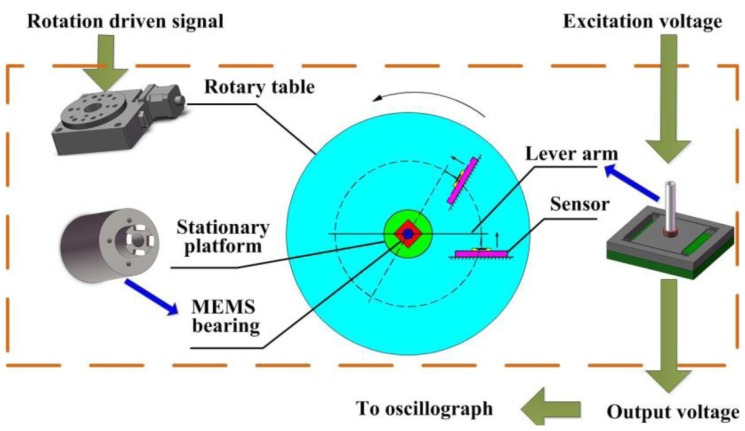
The system for friction measurement in MEMS bearings.

The practical measurement system is sketched in Figure 14. The ultra-precision computer-controlled rotary table generates the original rotation by its step motor which is transmitted to the shaft of the bearings by the force sensor and a silicon-based lever arm. The lever arm is fabricated by laser scribing apparatus, and the final length is 50 mm, width is 1 mm and thickness is 400 μm. The lever arm is adhered into the groove of bearing shaft; the sensor probe is vertically pressured to the lever arm with a certain force arm length, and there is no adhesive between the probe tip and lever arm.

**Figure 15.**
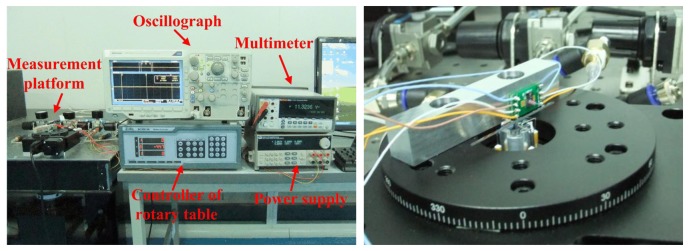
Photo of the assembled system (**left**) and the measurement platform (**right**).

**Figure 16.**
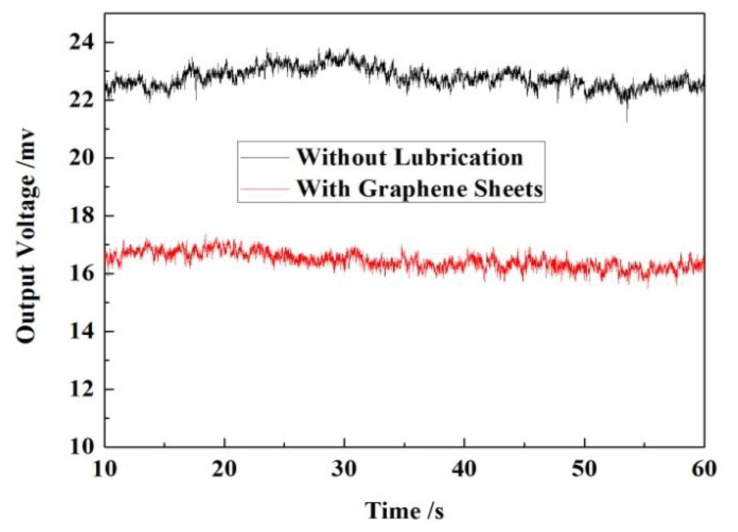
Curve obtained from the practical measurement.

The MEMS bearing is installed in the central hole of the rotary table by a stationary platform. The force sensor is adhered to an off-center settled fixture with an adjustable distance from the table center. The whole platform is fixed on a horizontal stand. The length of force arm is set as 20 mm which can be changed by moving the connection point between the lever arm and sensor probe. The sensor output is recorded by oscillograph when rotating the bearings. The assembled system is shown in Figure 15. The output voltage of the sensor was recorded by the oscillograph when the rotation was stable. In the measured curve, as shown in Figure 16, the black line is the result without any lubrication and the red line result was obtained after the graphene sheets were assembled onto the shaft surface by LB (Langmuir-Blodgett) technique [26,27]. The results indicate that the measured friction in the graphene sheets-assembled bearing is much lower than the one without any lubrication. We can collect the useful information of friction status by further processing the output signal of force sensors.

## 6. Conclusions

A force sensor with slotted-quad-beam structure has been designed, fabricated and tested for application in a friction measurement system. The proposed sensor structure uses stress concentration slots to enlarge the localized stress when the force is applied. A hollow stainless steel probe is bonded to the fabricated sensor chip by a tailor-made organic glass fixture. According to the results of sensor calibration, the sensitivity, maximum non-linearity error and measurement accuracy of the developed sensor are 0.813 mV/(V·mN), 1.57% FS and 2.03% FS, respectively. Compared with some force sensors described in the previous literature, the devised device exhibits a favorable performance in sensitivity, measurement range and overload protection. The bonded glass wafer gives the sensor a ten-fold overload protection over the allowed range. Practical experiments for the estimation of friction state in the bearings are conducted, and the results show the variation of friction in the bearings under different lubrication states, confirming the feasibility of utilizing the proposed force sensor to investigate the bearing friction. Our future work will be a follow-up study to develop a systematized sensor design process and further analyze the friction results.

## Figures and Tables

**Table 1.** Comparison with the existing devices.

	**This work**	**Ref. [23]**	**Ref. [25]**	**Ref. [14]**	**Ref. [24]**
Sensitivity (mV/V·mN)	0.813	4.1	0.11	0.1327	0.79
Measurement range (mN)	10	0.06	1 × 10^4^	120	25
Overload protection	Yes	Yes	Yes	No	No
